# Risk factors for HTLV-1 infection in Central Africa: A rural population-based survey in Gabon

**DOI:** 10.1371/journal.pntd.0006832

**Published:** 2018-10-12

**Authors:** Delia Doreen Djuicy, Augustin Mouinga-Ondémé, Olivier Cassar, Jill-Léa Ramassamy, Antony Idam Mamimandjiami, Rodrigue Bikangui, Arnaud Fontanet, Antoine Gessain

**Affiliations:** 1 Institut Pasteur, Unité d’Epidémiologie et Physiopathologie des Virus Oncogènes, Département de Virologie, Paris, France; 2 CNRS, UMR 3569, Paris, France; 3 Centre International de Recherches Médicales de Franceville, Groupe des Rétrovirus Animaux, BP: Franceville, Gabon; 4 Ecole Doctorale Régionale d’Afrique centrale, Infectiologie Tropicale, BP: Franceville, Gabon; 5 Institut Pasteur, Unité de Recherche et d’Expertise Epidémiologie des Maladies Emergentes, Département d’Infection et Epidémiologie, Paris, France; 6 Conservatoire National des Arts et Métiers, Unité PACRI, Paris, France; Hospital Universitário Professor Edgard Santos, BRAZIL

## Abstract

**Background:**

Human T-Lymphotropic Virus type 1 (HTLV-1) is a human oncoretrovirus that infects at least 5 to 10 million people worldwide and is associated with severe diseases. Africa appears as the largest HTLV-1 endemic area. However, the risk factors for the acquisition of HTLV-1 remain poorly understood in Central Africa.

**Methods:**

We conducted an epidemiological survey between 2013 and 2017, in rural areas of 6 provinces of Gabon, in a rainforest environment. Epidemiological data were obtained and blood samples were collected after informed consent. Plasma were screened for HTLV-1 antibodies by ELISA and the positive samples were then tested by Western blot (WB). Genomic DNA derived from buffy-coat was subjected to two semi-nested PCRs amplifying either HTLV-1 *env* gene or LTR region fragments.

**Results:**

We recruited 2,060 individuals over 15 years old, including 1,205 men and 855 women (mean age: 49 years). Of these, 299 were found to be ELISA HTLV-1/2 seropositive. According to WB criteria, 136 were HTLV-1 (6.6%), 25 HTLV-1/2 (1.2%) and 9 HTLV seroreactive (0.4%). PCR results showed that 146 individuals were positive for at least one PCR: 104 for the *env* gene and 131 for the LTR region. Based on both serological and molecular results, 179 individuals were considered infected with HTLV-1, leading to an overall prevalence of 8.7%. The distribution of HTLV-1 infection was heterogeneous across the country. Based on multivariable analyses, female gender, increasing age, ethnicity (Pygmy) and multiple hospitalizations (more than 5 times) were found to be independent risk factors for HTLV-1 infection. Furthermore, a non-human primate bite appeared to be marginally associated with a higher risk of HTLV-1 infection.

**Conclusion:**

Based on state-of-the-art serological and molecular methods, we have demonstrated that rural adult populations in Gabon are highly endemic for HTLV-1. Our results regarding risk factors should lead to public health actions aiming to reduce HTLV-1 transmission.

## Author summary

Human T-Lymphotropic Virus type 1 (HTLV-1) infects at least 5 to 10 million people worldwide and is associated with severe diseases. Africa, particularly the sub-Saharan region, is considered as the largest HTLV-1 endemic area. HTLV-1 can be acquired through three main routes: mother-to-child, sexual and through contaminated blood products. In addition, zoonotic transmission from non-human primates still occurs in specific rural African populations. Gabon is considered to be a very high HTLV-1 endemic area. However, risk factors for such a retroviral infection remain poorly known in Central Africa. In this study, using state-of-the-art serological and molecular tests, we aimed to get new insights on risk factors associated with HTLV-1 infection in rural Gabon. We report an overall prevalence of HTLV-1 of 8.7%, demonstrating that HTLV-1 is highly endemic in rural adult Gabonese populations. In the study population, we identified female gender, increasing age, ethnicity (Pygmy) and multiple hospitalizations (more than 5 times) as independent risk factors for HTLV-1 infection. Furthermore, a non-human primate bite appeared to be marginally associated with a higher risk of HTLV-1 infection. Our results regarding the risk factors for such a retroviral infection are important for public health actions aiming to reduce HTLV-1 transmission.

## Introduction

Human T-lymphotropic virus type 1 (HTLV-1), the first human retrovirus discovered [1], is the etiological agent of several pathologies, mainly a very severe T-cell lymphoproliferation named Adult T-Cell Leukemia Lymphoma (ATLL) and a chronic disabling neuro-myelopathy, the Tropical Spastic Paraparesis/HTLV-1 Associated Myelopathy (TSP/HAM) [1–4]. HTLV-1 is not ubiquitously distributed worldwide. Indeed, it is mainly present in foci where viral prevalence can reach 2 to 40% in adults, depending on age, sex and geography. The most important HTLV-1 endemic areas are: the Southern part of the Japanese archipelago, several areas in South America and the Caribbean basin as well as some areas of Australo-Melanesia, Iran and large regions of sub-Saharan Africa. This human oncoretrovirus is estimated to infect at least 5 to 10 million people worldwide [5, 6]. While the great majority of HTLV-1 infected individuals remains asymptomatic throughout their life, ATLL and TSP/HAM occur in 2 to 7% of them [7]. The three main routes of HTLV-1 transmission are: mother-to-child through prolonged breastfeeding (mostly over 6 months) [8], sexual (mainly from male to female) [9] and by blood products contaminated with infected cells [10]. HTLV-1 originates from its simian counterpart STLV-1, which is highly prevalent in several Non-Human Primates (NHPs) species. Zoonotic transmission of STLV-1 still occurs mainly through severe NHP bites, at least in Central and West Africa [11, 12].

Sub-Saharan Africa is considered as the largest HTLV-1 endemic area accounting for at least half of the infected individuals worldwide (2.5 to 5 millions). However, the situation of HTLV-1 in Africa is not well known. Indeed, the majority of previous studies have been carried out either on very specific populations such as pregnant women, blood donors or hospitalized patient series, or in heterogeneous and relatively small groups of rural or urban inhabitants of a specific town or village or area [5, 13]. All these groups are far from being representative of the population of a given region or country. Furthermore, many of these prevalence studies only include serological analyses, without molecular detection of HTLV-1 proviral DNA. Lastly, there are still few epidemiological studies investigating HTLV-1 acquisition risk factors in the African continent [14–17]. Gabon, located in Central Africa, appears to be highly endemic for HTLV-1 and is an appropriate area for the study of HTLV-1 acquisition risk factors [18–21]. Indeed, to date, no public health action has been undertaken to prevent dissemination of this virus in Gabon, while the four modes of transmission are still prevalent in this country.

In this work, we performed a large epidemiological study among adult inhabitants of rural Gabon. We used state-of-the-art assays for both serological and molecular diagnoses. Our first objective was to provide better knowledge of the prevalence and the geographical distribution of HTLV-1 infection in Gabon. The second was to get new insights into the risk factors for the acquisition of HTLV-1 among adults living in this highly endemic area.

## Material and methods

### Study population

Between 2013 and 2017, we conducted epidemiological surveys involving inhabitants of rural areas living mainly in a rainforest environment, in 6 out the 9 provinces of Gabon. A systematic approach for the enrolment of adults (over 15 years old) was carried out in the populations from all reachable villages and settlements, scattered along side roads and tracks across the primary tropical forest. We included all volunteers. A standardized questionnaire was used to collect epidemiological data. Besides demographic information, we focused our questions on the potential risk factors for HTLV-1 acquisition including scarification, hospitalization, transfusion and contacts with wild animals and bush meat during hunting and/or butchering activities (especially with monkeys and apes). A 5 to 10 ml blood sample was collected on EDTA from all consenting individuals. Plasma and buffy coat were obtained 48 to 72 h after sampling and kept frozen at -80°C.

### Ethics

Ethical approval was obtained from the Comité National d’Ethique of Gabon (Permit number PROT 0011/2013/SG/CNE). Prior to field sampling, individual written informed consent was obtained from all participants after detailed information and explanations of the study were provided to the community. Written informed consent for children was obtained from their parents or recognized guardians.

### HTLV-1 serological tests

Plasma samples were screened for HTLV-1/2 antibodies by ELISA tests (HTLV-I/II ELISA 4.0, MP Biomedicals), and confirmatory Western blot assays (HTLV BLOT 2.4, MP Biomedicals) for all ELISA positive samples. Results were interpreted according to the manufacturer’s instructions. Thus, the samples were classified as HTLV-1 seropositive, HTLV-2 seropositive, both HTLV-1/2 seroreactive, HTLV seroreactive, indeterminate and seronegative.

### HTLV-1 molecular tests

High molecular weight DNA was extracted from peripheral blood buffy coats (PB-BC) using the QIAamp blood minikit (Qiagen). DNA samples were determined as amplifiable after a polymerase chain reaction (PCR) positive result, performed on the human β-globin gene. One microgram of each DNA sample was then tested by two different semi-nested PCRs: according to protocols previously described [22]: a first one to amplify a 522-bp fragment of the *env* gene, and a second one to amplify a 450-bp fragment of the LTR-3’ region of HTLV-1. A sample was considered as PCR positive when a band of the expected size was clearly detected on agar gel.

### Positive criteria for HTLV-1 infection

An individual was considered as infected with HTLV-1 if the WB profile was either HTLV-1, or HTLV-1/2 or HTLV. Furthermore, an individual with an indeterminate WB profile associated with at least one HTLV-1 positive PCR, was also considered as HTLV-1 infected.

### Statistical methods

Age was compared between Pygmy and Bantu ethnic groups using the Student *t* test. HTLV-1 infection prevalence was compared between provinces using the Chi-square test, and between age groups using the Chi-square test for trends. Logistic regression was used to identify factors associated with HTLV-1 infection and to estimate odds ratios (OR) and 95% confidence intervals (95%CI). ORs were presented as crude or age and gender adjusted (univariable analysis) and all variables adjusted (multivariable analysis). Variables retained in the final model were considered statistically significant if *p* values were < 0.05, and marginally significant if *p* values were < 0.07. Multiple imputations were performed to replace missing values for the “number of hospitalizations” variable, using the multivariable imputation by chained equations (MICE) method [23]. All analyses were performed using STATA 15.0 software (Stata Corporation, College Station, TX, USA).

## Results

**Fig 1:**
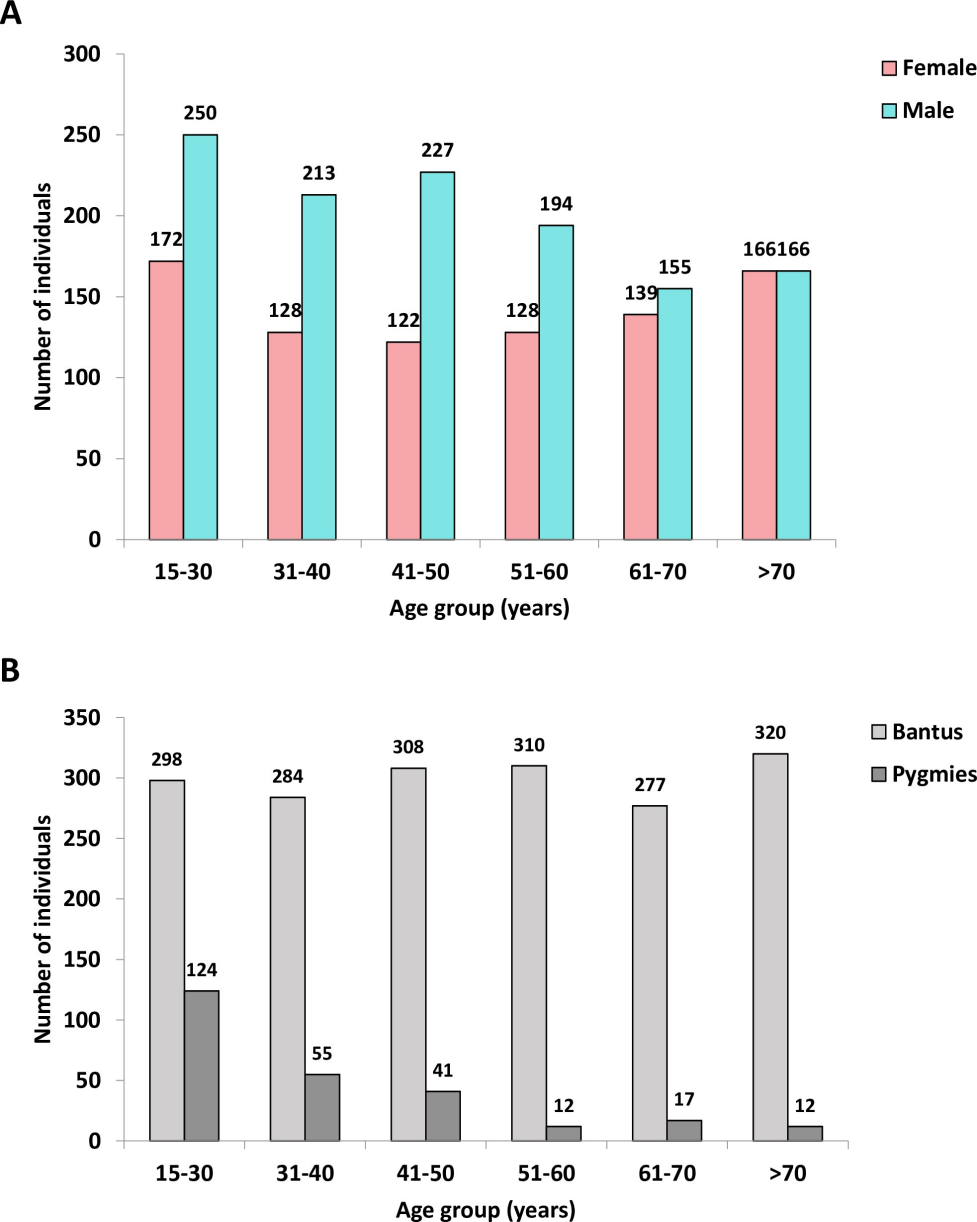
Age distribution according to gender (A) and ethnic group (B).

We recruited 2,060 individuals (older than 15), including 1,205 men and 855 women (median age: 48, interquartile range: 33–64), originating from the 6 investigated provinces of Gabon (Fig 1A). A large majority of them (1,797) belonged to the Bantu ethnic group, and 261 belonged to the Pygmy group (Fig 1B). Of note, the Pygmy group included younger individuals than the Bantu one (mean age 37 years *vs* 51, p<0.001).

### HTLV-1 serological results

Among the 2,060 plasma samples tested by ELISA, 299 were found HTLV-1/2 seropositive. Using WB criteria provided by the manufacturer, 136 were HTLV-1 seropositive (6.6%), 25 HTLV-1/2 (1.2%), 10 HTLV-2 (0.5%), 9 HTLV seroreactive (0.4%) and 85 sero-indeterminate (4.1%). Moreover, 34 were seronegative. Based on strict WB results as defined by the manufacturer’s assay (HTLV Blot 2.4, MP Biomedicals) and including only HTLV-1 and HTLV-1/2 profiles, the overall HTLV-1 seroprevalence was 7.8% (95%CI 6.7–9.1, 161/2,060) (Table 1).

**Table 1:** PCR results according to the HTLV Western blot profiles.

WB results		PCR results				
WB profiles	Number of WB profile (N)	*env*	LTR-3’	*env* and LTR-3’	*env* or LTR-3’ (n)	% (n/N)
**HTLV-1**	**136**	**77**	**101**	**67**	**111**	**82** **(111/136)**
**HTLV-2**	**10**	**0**	**0**	**0**	**0**	**0** **(0/10)**
**HTLV-1/2**	**25**	**15**	**21**	**15**	**21**	**84** **(21/25)**
**HTLV**	**9**	**4**	**3**	**2**	**5**	**55.6** **(5/9)**
**INDETERMINATE**	**85**	**8**	**6**	**5**	**9**	**10.6** **(9/85)**
**NEGATIVE**	**34**	**0**	**0**	**0**	**0**	**0** **(0/34)**
**Total**	**299**	**104**	**131**	**89**	**146**	**49** **(146/299)**

%: proportion of at least one PCR positive result (*env* gene or LTR-3’ region) according to WB profiles

### HTLV-1 molecular detection results

Molecular amplification was performed on DNA extracted from the PB-BC of 299 ELISA positive individuals. The results showed that 146 were positive for at least one PCR (104 for the *env* gene, 131 for the LTR region), and 89 for both PCRs (Table 1).

### HTLV-1 prevalence in the studied population

**Fig 2:**
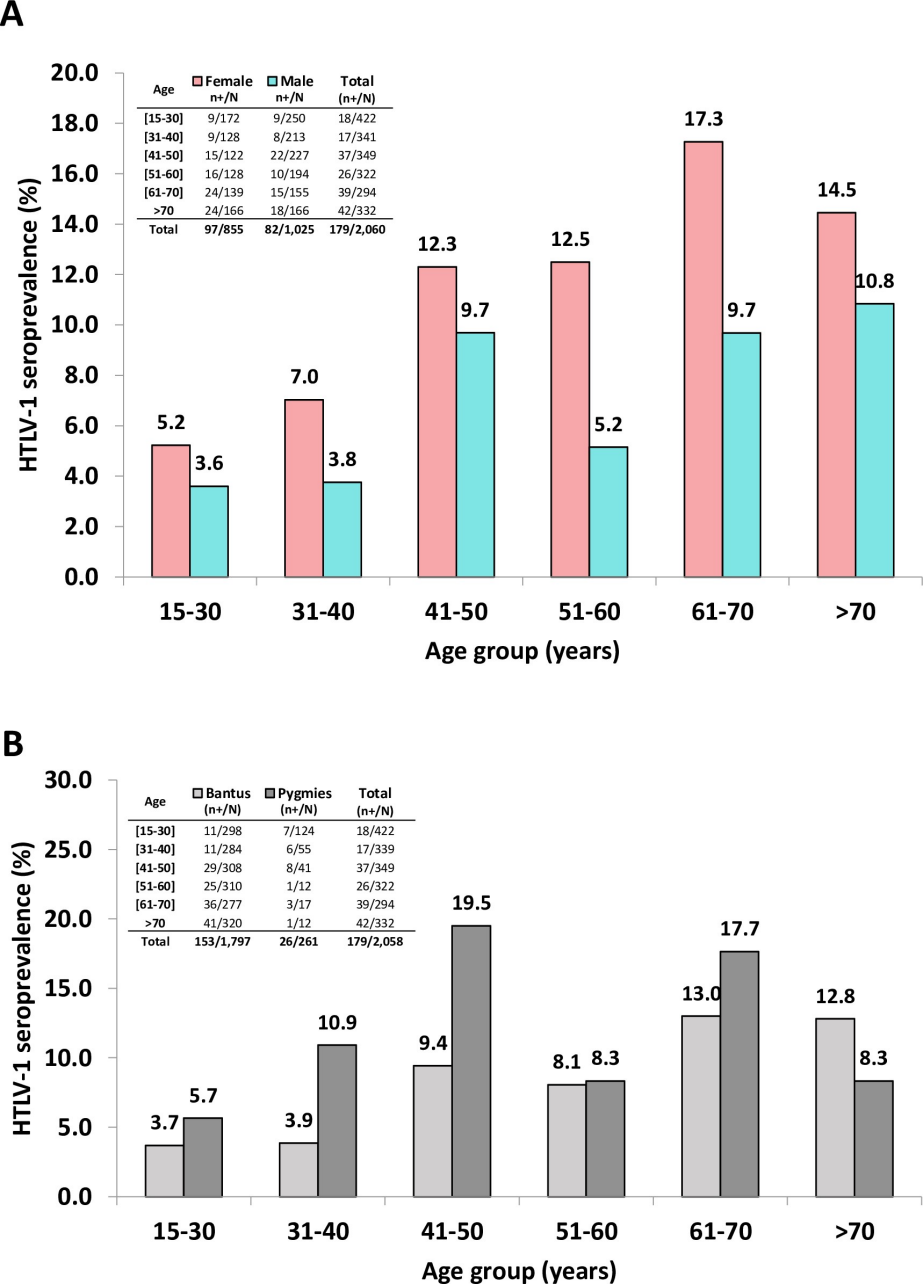
HTLV-1 seroprevalence according to age and sex (A), and ethnic group (B).

Based on both serological and molecular results, 179 individuals were considered as HTLV-1 infected. This includes: i) the 170 individuals with a WB profile being either HTLV-1, HTLV-1/2 or HTLV, and ii) the 9 individuals with an indeterminate WB profile associated with at least one positive HTLV-1 PCR test (Table 1). Thus, 179 HTLV-1 infected persons were included in the epidemiological analysis (Fig 2A and 2B). This leads to an overall HTLV-1 infection prevalence of 8.7% (95%CI 7.5–10.0).

### Risk factors for HTLV-1 infection in the studied population

Univariable analysis revealed that women are significantly more likely to be HTLV-1 infected than men (age-adjusted OR 1.69; 95%CI 1.2–2.3, Table 2). Indeed, 97 of 855 women (11.35%) and 82 of 1,205 men (6.8%) were HTLV-1 infected. As illustrated (Fig 2A and Table 2), the age specific seroprevalence gradually increases in the study population (p <0.001), reaching 17.3% among women of the 61 to 70 age group. Furthermore, Pygmy individuals are at increased risk of being HTLV-1 seropositive compared to Bantu people, after adjustment for age and sex (Adjusted OR 1.67; 95%CI 1.04–2.68, Table 2 and Fig 2B).

**Table 2:** Univariable analysis of risk factors associated with HTLV-1 infection in the study population.

	HTLV-1 results		Crude OR (95% CI)	P-value	Adjusted OR^a^ (95% CI)	P-value
	Negative	Positive (%)				
**Gender**						
**M**	1,123	82 (6.80)	1		1	
**F**	758	97 (11.35)	1.75 (1.29-2.39)	0.0003***	1.69 (1.23-2.3)	0.001***
**Age**						
**15-30**	404	18 (4.27)	1	<0.001***	1	<0.001***
**31-40**	324	17 (4.99)	1,18 (0.60-2.32)		1.20 (0.61-2.37)	
**41-50**	312	37 (10.60)	2.66 (1.49-4.76)		2.76 (1.54-4.95)	
**51-60**	296	26 (8.07)	1.97 (1.06-3.66)		1.99 (1.07-3.7)	
**61-70**	255	39 13.27)	3.43 (1.92-6.13)		3.34 (1.87-5.97)	
**>70**	290	42 (12.65)	3.25 (1.83-5.76)		3.11 (1.75-5.53)	
**Missing**	0	0 (0)	-	-	-	-
**Ethnic group**						
**Bantus**	1,644	153 (8.51)	1		1	
**Pygmies**	235	26 (9.96)	1.19 (0.77-1.84)	0.4382	1.67 (1.04-2.68)	0.034*
**Missing**	2	0 (0)	-	-	-	-
**Province**						
**Nyanga**	171	10 (5.52)	1	0.027**	1	0.013**
**Ngounié**	334	31 (8.49)	1.59 (0.76-3.31)		1.48 (0.7-3.11)	
**Haut-Ogooué**	494	52 (9.52)	1.8 (0.89-3.62)		2.09 (1.03-4.24)	
**Ogooué-Lolo**	228	28 10.94)	2.1 (0.99-4.44)		2.24 (1.05-4.78)	
**Ogooué-Ivindo**	129	21 (14.00)	2.78 (1.27-6.12)		3.54 (1.59-7.87)	
**Woleu-Ntem**	525	37 (6.58)	1.21 (0.59-2.47)		1.58 (0.76-3.3)	
**Missing**	0	0 (0)	-	-	-	-
**Hunting (among men)**						
**No**	273	14 (4.88)	1		1	
**Yes**	841	68 (7.48)	1.58 (0.87-2.85)	0.1284	1.34 (0.75-2.41)	0.321
**Missing**	9	0 (0)	-	-	-	-
**Hunting NHP (among men)**						
**No**	363	22 (5.71)	1		1	
**Yes**	689	58 (7.76)	1.39 (0.84-2.31)	0.20	1.21 (0.72-2.01)	0.472
**Missing**	71	2 (2.74)	-	-	-	-
**Keeping NHP as pets**						
**No**	1,712	161 (8.60)	1		1	
**Yes**	134	15 (10.07)	1.19 (0.68-2.08)	0.54	1.46 (0.82-2.6)	0.193
**Missing**	35	3 (7.89)	-	-	-	-
**Bitten by a NHP**						
**No**	1,824	171 (8.57)	1		1	
**Yes**	45	8 (15.09)	1.89 (0.87-4.08)	0.097	2.31 (1.05-5.11)	0.038*
**Missing**	12	0 (0)	-	-	-	-
**Butchering**						
**No**	264	26 (8.97)	1			
**Yes**	1,298	124 (8.72)	0.99 (0.99-1.0)	0.795	1.00 (0.99-1.01)	0.674
**Missing**	319	29 (8.33)	-	-	-	-
**Hospitalization**						
**No**	699	62 (8.15)	1		1	
**Yes**	1,175	116 (8.99)	1.11 (0.81-1.54)	0.5148	0.94 (0.68-1.31)	0.730
**Missing**	7	1 (12.50)	-	-	-	-
**Number of hospitalization**						
**0**	575	41 (6.66)	1	0.005**	1	0.109
**1**	537	43 (7.41)	1.12 (0.72-1.75)		1.02 (0.65-1.61)	
**2-5**	547	64 (10.47)	1.64 (1.09-2.47)		1.30 (0.86-1.99)	
**>5**	71	15 (17.44)	2.96 (1.56-5.62)		2.27 (1.18-4.37)	
**Missing**	151	16 (9.58)	1.49 (0.81-2.72)		1.36 (0.74-2.51)	
**Transfusion**						
**No**	1,658	148 (8.19)	1		1	
**Yes**	209	30 (12.55)	1.61 (1.06-2.44)	0.026*	1.35 (0.88-2.07)	0.165
**Missing**	14	1 (6.67)	-	-	-	-
**Circumcision (among men)**						
**No**	41	3 (6.82)	1		1	
**Yes**	1,079	79 (6.82)	1.00 (0.30-3.30)	0.999	1.19 (0.35-3.95)	0.777
**Missing**	3	0 (0)	-	-	-	-
**Scarification**						
**No**	682	50 (6.83)	1		1	
**Yes**	1,180	126 (9.65)	1.46 (1.04-2.05)	0.031*	1.23 (0.87-1.75)	0.242
**Missing**	19	3 (13.64)	-	-	-	-
**Tattoo**						
**No**	1,553	154 (9.02)	1		1	
**Yes**	306	21 (6.42)	0.69 (0.43-1.11)	0.127	0.96 (0.59-1.57)	0.880
**Missing**	22	4 (15.38)	-	-	-	-

*p<0,05

**p<0,01

***p<0,001

^a^ Adjusted OR on age and sex

**Fig 3:**
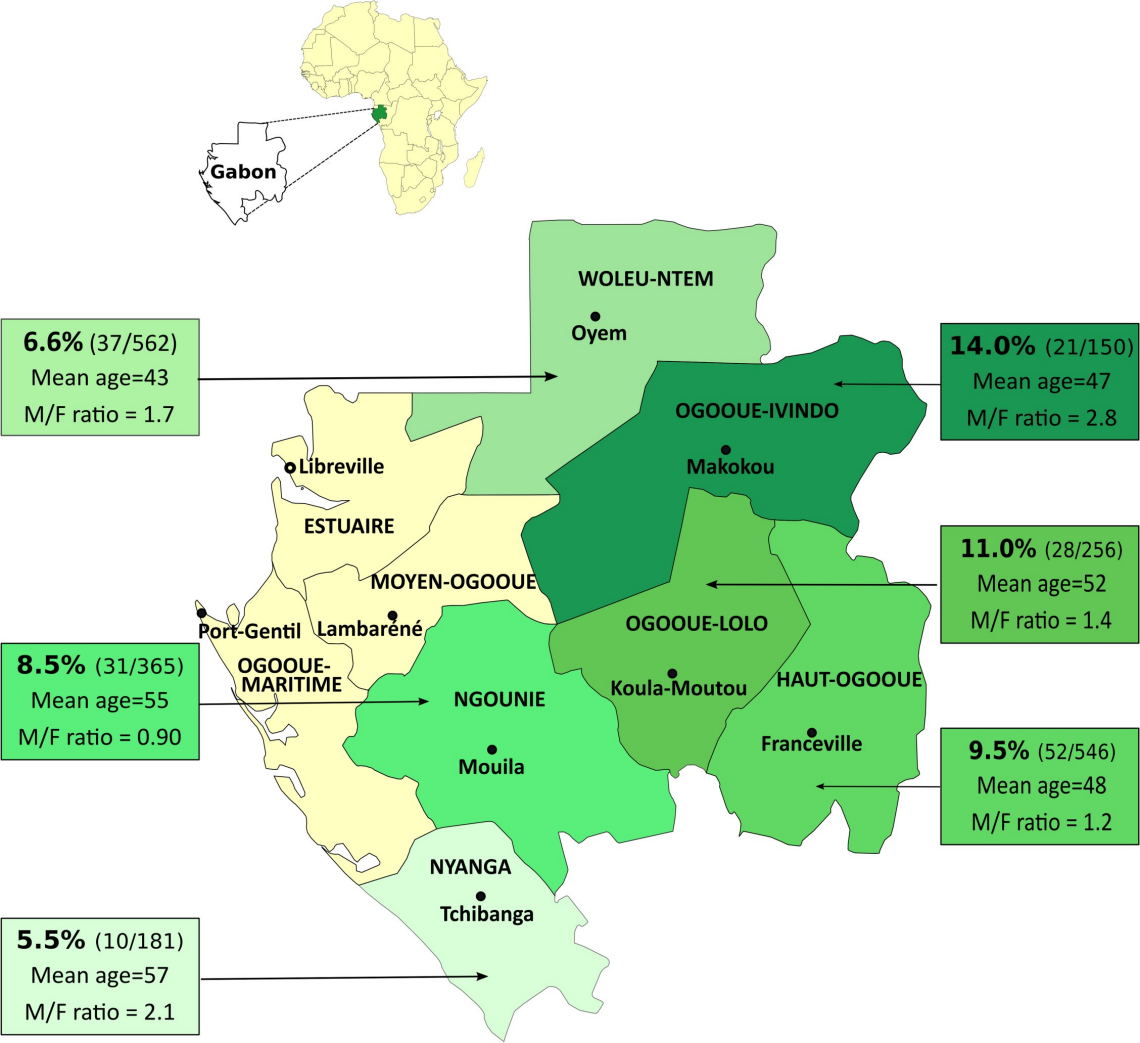
Prevalence of HTLV-1 according to the geographical distribution of the six provinces studied in Gabon. The data given in the figure indicate, for each province, the HTLV-1 prevalence, the number of positive individuals among those tested, the mean age and the sex ratio. This map was modified from https://commons.wikimedia.org/wiki/Atlas_of_Gabon using Inkscape 0.92.2.

Individuals included in this study originate from 6 of the 9 Gabonese provinces (Fig 3). HTLV-1 seroprevalence varies according to the geographic origin of infected individuals (Fig 3 and Table 2). Ogooué-Ivindo and Ogooué-Lolo provinces present a significantly higher HTLV-1 prevalence (respectively 14% and 11%; p = 0.027) compared to Ngounié, Woleu-Ntem and Nyanga provinces, where lower infection rates were recorded. Moreover, the Haut-Ogooué province shows an intermediate HTLV-1 seroprevalence (9.5%), which is not significantly different from the other provinces (Fig 3 and Table 2).

Indicators for HTLV-1 parenteral transmission were also assessed. According to crude OR, HTLV-1 infection is associated with behaviors leading to scarifications (crude OR 1.46; 95%CI 1.04–2.05), an history of blood transfusion (crude OR 1.61; 95%CI 1.06–2.44), and an increased number of hospitalization (more than five times, crude OR 2.96; 95%CI 1.56–5.62), but not with tattoo nor circumcision. However, when adjusted for age and sex, only individuals with 5 hospitalizations or more were at increased risk of being HTLV-1 infected (Table 2).

Since our study was conducted in primary tropical forest areas and focused on individuals potentially at risk of acquiring HTLV-1 through interspecies transmission, we also obtained information on contacts with NHPs. No association was observed with hunting and butchering activities or keeping NHPs as pets. However, a higher risk was observed among individuals who reported having been bitten by a NHP. Indeed, 8 of 53 individuals (15.1%) were HTLV-1 seropositive (adjusted OR 2.31; 95%CI 1.05–5.11, Table 2).

In the final multivariable analysis model (Table 3), we found an increased independent risk of HTLV-1 infection in women (OR 1.61; 95%CI 1.16–2.23), the elderly people (e.g. >70; OR 3.47; 95%CI 1.89–6.37), persons having a history of multiple hospitalizations (more than 5 times, OR 2.38; 95%CI 1.21–4.66), and individuals belonging to the Pygmy ethnic group (OR 1.93; 95%CI 1.18–3.14). In addition, a NHP bite appears to be marginally associated with a higher risk of HTLV-1 infection (OR 2.16; 95%CI 0.97–4.8).

**Table 3:** Multivariable analysis of risk factors associated with HTLV-1 infection in the study population.

Risk factors	OR (95% CI)	P-value
**Gender**		
**Male**	1	0.004**
**Female**	1.61 (1.16-2.23)	
**Age group**		
**15-35**	1	<0.001***
**31-40**	1.28 (0.64-2.54)	
**41-50**	3.03 (1.66-5.53)	
**51-60**	2.25 (1.17-4.31)	
**61-70**	3.75 (2.04-6.91)	
**>70**	3.47 (1.89-6.37)	
**Number of hospitalization**		
**0**	1	0.05
**1**	1.03 (0.66-1.6)	
**2-5**	1.35 (0.87-2.1)	
**>5**	2.38 (1.21-4.66)	
**Ethnic Group**		
**Bantus**	1	0.008**
**Pygmies**	1.93 (1.18-3.14)	
**Bitten by a NHP**		
**No NHP Bite**	1	0.06
**NHP Bite**	2.16 (0.97-4.8)	

**p<0.01

***p<0.001

## Discussion

This study represents one of the largest epidemiological study conducted in Central Africa. We show that HTLV-1 is highly endemic among villagers living in the different provinces of the primary tropical forest of Gabon. These data confirm the high prevalence of HTLV-1 in Gabon and extend it to most rural areas, since we studied adult populations of both sexes, from 6 of the 9 rural provinces of this country. Previous studies mainly carried out in pregnant women or in populations from some regions of Gabon (mostly Franceville area and/or Eastern Gabon regions), have observed a high level of HTLV-1 infection [18–21, 24, 25]. However, such studies were mostly based on serological analyses with, for the oldest studies, Western blot interpretation criteria that are now considered as poorly specific. The results of our paper confirm the low specificity of the ELISA tests used for HTLV-1 serological screening. Furthermore, most of the indeterminate WB profiles (89.4% in our study) were PCR negative in DNA extracted from blood. These findings are frequently observed in central Africa where some of these false positives correspond to non-specific cross-reactivities, particularly between HTLV-1 Gag proteins and antigens derived from malaria [26]. Here, on the basis of state-of-the-art serological and molecular assays, we confirm a high level of retroviral infection. Surprisingly, this prevalence (8.7%) appears to be higher than in neighboring countries. Indeed, if we analyze the results obtained on similar populations (pregnant women or Bantu adults from rural areas of the rainforest), and using comparable assays, it is obvious that the overall level of HTLV-1 prevalence is at least 2 to 3 times higher in Gabon than in South Cameroun, in Congo or in Central African Republic [27–29]. The reasons for these differences remain to be elucidated. Of note, the existence of high prevalence foci close to low or very low endemic areas is a hallmark of HTLV-1 epidemiology. In South Japan, areas of low HTLV-1 prevalence can be very close to highly endemic areas, especially in rural regions. Furthermore, in a previous study performed in a geographically limited area of Eastern Gabon, the HTLV-1 seroprevalence rates greatly varies between villages, from 5 to 17.8% [21].

In our study, we observed a significant increase in HTLV-1 infection with age, as well as a higher prevalence in women. Such findings are well-known major characteristics of HTLV-1 epidemiology, regardless the endemic level and the studied areas. For instance, this has already been observed in rural populations of Guinea Bissau [15, 17], as well as in patients hospitalized in Cameroon [28] or in Benin [30]. Lastly, this increased prevalence in women and with age is commonly found in various populations, such as inhabitants of Salvador de Bahia in Brazil, workers in Jamaica [31] or urban inhabitants of Southern Japan [32]. Such consistency indicates that this is a major specificity of this virus. It has been considered that cumulative sexual transmission over the years, predominantly occurring from male to female, may explain this specificity [33]. In some cases, a cohort effect can also be partly responsible for such an increase with age, as demonstrated in blood donors in Japan [34]. Surprisingly, a higher HTLV-1 seroprevalence in women was not observed in two previous studies performed in Gabon [19, 35]. These discrepancies might be related to the age of the studied population. Indeed, significant sex-specific differences in HTLV-1 prevalence are not found in young adult age groups [21, 32, 33, 36].

Despite the small number of Pygmy individuals included in this study as compared to Bantu (261 *vs* 1,797), the prevalence of HTLV-1 is significantly higher in Pygmies. Besides, in our multivariate analysis, being a Pygmy was associated with an increased independent risk of HTLV-1 infection (OR 1.93; 95%CI 1.18–3.34). Such findings were previously observed for HTLV-1 and also for HTLV-2 infection in Bakola’s Pygmies living in the Ocean region, located in the Western part of South Cameroon [37]. The reasons why these retroviral infections have a higher endemic level in Pygmy populations remain unclear. It has been hypothesized that it may be linked to a founder effect with persistent high level of viral transmission. This may either be due to specific intrafamilial activities (for example prolonged breastfeeding or breastfeeding of several children by the same woman…), or to behavioral factors such as initiation rites, leading to blood contamination by non-sterile needles or sharp objects such as razor blades. Some of these hypotheses have also been proposed for other populations highly endemic for HTLV-1 or HTLV-2, such as Indigenous Australians living in Central Australia [38] or Amerindians from South American countries [39].

Concerning the distribution of HTLV-1 prevalence in the different provinces of Gabon, our data indicate a heterogeneous distribution ranging from 5.5% to 14%. Contrary to what was previously suggested, we have not observed a gradient of HTLV-1 prevalence following the North-South axis. Such discrepancy could be related to various factors including the non-homogeneous and therefore non-comparable structure of the populations previously studied (urban *vs* rural). In a previous study, the HTLV-1 age-adjusted prevalence rate was significantly higher in rural areas of Gabon than in urban areas [19]. Moreover, the gradient was especially suggested when looking at the population of pregnant women living in urban areas [24].

In this study, 15% of inhabitants bitten by a NHP were HTLV-1-infected compared to only 8.5% for people not bitten by such primates. In addition, NHP bite appears to be marginally associated with a high risk of HTLV-1 infection in our multivariable analysis. These results are reminiscent of those recently published that demonstrated that the bite by a monkey is a risk factor for HTLV-1 infection in a population of hunters living in tropical forest villages of Southern Cameroon [12]. They also echo the recent case-report of a young girl from a village in Eastern Gabon, which showed likely interspecies transmission of STLV-1 upon a severe bite by a *C*. *nictitans* [40]. Lastly, several publications also indicate that simian foamy viruses (belonging to another family of simian retroviruses) are highly endemic in Central African monkeys and apes, and can easily be transmitted to humans by severe bites from a NHP, leading to chronic infection [41].

In our study, one other risk factor associated with HTLV-1 is hospitalization. Indeed, HTLV-1 prevalence increases from 6% to 17% with the number of hospital stays. In the multivariable analysis, there is an increased independent risk of HTLV-1 infection in individuals with a history of multiple hospitalizations (more than 5 times). The reasons for the existence of such a risk are not clear. It could be linked to the nosocomial acquisition of this retroviral infection in hospital, either by transfusion of cells infected with HTLV-1, or by contamination through the use of infected syringes or non-sterile utensils. However, transfusion is not associated with HTLV-1 infection in our model. One may suppose that HTLV-1 is not actually transmitted at the hospital. For instance, the virus could be acquired through severe bite, which could secondarily lead to hospitalization. Similarly, a given illness could lead the patient to first visit a traditional healer with risky practices (e.g. use of non sterile material), before heading to more classical hospitalization.

We hoped that this study would alert Gabonese public health authorities and promote appropriate measures to prevent HTLV-1 infection throughout the country, at least in rural populations in which we reported high prevalence. Despite the fact that Gabon is both a highly HTLV-1 endemic area and a high development index country, it has not yet introduced routine screening for HTLV-1, especially among blood donors. Importantly, a previous study in Gabon has already demonstrated that transfusion is a major risk factor for HTLV-1 acquisition in children [16]. In addition, several studies around the world have clearly demonstrated that HAM/TSP is frequently linked to HTLV-1 acquisition of blood by transfusion of contaminated cells [42]. Routine testing of blood donors seems the most necessary and easiest measure to implement quickly. In this context, we are currently conducting a survey, in close collaboration with the Libreville blood bank, in order to get new insights in the prevalence of HTLV-1 among blood donors. Another control measure that could be implemented is the screening for HTLV-1 infection in pregnant women. However, this raises the delicate and complex issue of decreasing or stopping breastfeeding in HTLV-1 infected women in a context of resource-limited countries. Finally, public health information campaigns on the zoonotic risks associated with hunting monkeys are also necessary.

## Supporting information

S1 Checklist STROBE checklist for cross-sectional study. (DOC)
